# Examining Analytic Practices in Latent Dirichlet Allocation Within Psychological Science: Scoping Review

**DOI:** 10.2196/33166

**Published:** 2022-11-08

**Authors:** Lauryn J Hagg, Stephanie S Merkouris, Gypsy A O’Dea, Lauren M Francis, Christopher J Greenwood, Matthew Fuller-Tyszkiewicz, Elizabeth M Westrupp, Jacqui A Macdonald, George J Youssef

**Affiliations:** 1 Centre for Social and Early Emotional Development (SEED) School of Psychology Deakin University Geelong Australia; 2 Centre for Adolescent Health Murdoch Children’s Research Institute Melbourne Australia; 3 Department of Paediatrics University of Melbourne Melbourne Australia; 4 Judith Lumley Centre La Trobe University Melbourne Australia

**Keywords:** latent Dirichlet allocation, LDA, review, analysis, methodology

## Abstract

**Background:**

Topic modeling approaches allow researchers to analyze and represent written texts. One of the commonly used approaches in psychology is latent Dirichlet allocation (LDA), which is used for rapidly synthesizing patterns of text within “big data,” but outputs can be sensitive to decisions made during the analytic pipeline and may not be suitable for certain scenarios such as short texts, and we highlight resources for alternative approaches. This review focuses on the complex analytical practices specific to LDA, which existing practical guides for training LDA models have not addressed.

**Objective:**

This scoping review used key analytical steps (data selection, data preprocessing, and data analysis) as a framework to understand the methodological approaches being used in psychology research using LDA.

**Methods:**

A total of 4 psychology and health databases were searched. Studies were included if they used LDA to analyze written words and focused on a psychological construct or issue. The data charting processes were constructed and employed based on common data selection, preprocessing, and data analysis steps.

**Results:**

A total of 68 studies were included. These studies explored a range of research areas and mostly sourced their data from social media platforms. Although some studies reported on preprocessing and data analysis steps taken, most studies did not provide sufficient detail for reproducibility. Furthermore, the debate surrounding the necessity of certain preprocessing and data analysis steps is revealed.

**Conclusions:**

Our findings highlight the growing use of LDA in psychological science. However, there is a need to improve analytical reporting standards and identify comprehensive and evidence-based best practice recommendations. To work toward this, we developed an LDA Preferred Reporting Checklist that will allow for consistent documentation of LDA analytic decisions and reproducible research outcomes.

## Introduction

### Background

The past 25 years have seen an enormous increase in the availability of so called “big data,” a broad term describing very large, but typically unstructured data sets [1]. One example of big data is textual data, which describes any source of data that contains written words or words that are transcribed from speech. The big data era [1] has seen increasing availability of large textual data sets derived from a variety of sources including web-based forums (eg, Reddit), social microblogging platforms (eg, Twitter, Facebook, and Instagram), formal documentation (eg, discharge summaries and clinical notes), qualitative data sets, Google Books, and scientific literature. Big data sets have been used in a variety of research areas such as travel [2], digital humanities [3], and marketing [4]. Given that textual data sets may provide important insights into trends and associations relating to human behavior and attitudes, it is not surprising that the use of these data sets is increasing in the psychological sciences.

Considering the potential size and complexity of big textual data sets, psychology researchers have begun to rely on natural language processing (NLP) techniques. These computational methods are used to analyze and represent written text [5,6]. Topic modeling approaches are largely automated and allow researchers to effectively and efficiently engage with big textual data sets in ways that cannot be practically achieved with nonautomated techniques for synthesizing (ie, literature reviews) and analyzing (ie, qualitative approaches) textual data.

There are a range of topic modeling approaches available [7]; for example, latent semantic analysis is a nonprobabilistic method that can be used to draw meaning from textual data [8], and Dirichlet multinomial mixture–based methods may perform better for smaller texts [9]. However, one commonly used NLP technique used in health research is latent Dirichlet allocation (LDA), which is a machine learning methodology that uses Bayesian probability–based algorithms to discover latent (unobserved) “topics” based on co-occurrence of words from within a body of text (ie, corpus). Although detailed explanations of these algorithms can be found in the studies by Blei et al [10] and Griffiths and Steyvers [11], in simple terms, LDA identifies latent topics within a corpus by estimating both *document-topic probabilities* (ie, the probability that each document is generated by any specific topic) and *word-topic probabilities* (ie, the probability that any word is generated by a specific topic; [12,13]). LDA assumes that documents comprise many latent topics and that latent topics comprise many words [12]. Briefly, the LDA algorithm first requires the user to specify the number of latent topics (*k*) expected within the corpus. Initially, the algorithm iterates through each document (ie, unit of text) and words within the document and randomly assigns the words to one of the latent topics. This results in a distribution of document-topic probabilities (ie, the probability of the words in any document assigned to each of the *k* topics) and word-topic probabilities (ie, the proportion of times a word has been assigned to each of the *k* topics) based on random allocation. This random allocation is then optimized by iterating through each document and words within the documents, recalculating the probability of a word belonging to a topic given a particular document, and then updating the word-topic probabilities across all documents. In addition to the number of topics (*k*), the LDA algorithm is influenced by 2 other parameters (also known as hyperparameters) that can be specified by the researcher and affects how topics are represented across documents and by words. Alpha influences how documents contribute to topics, with larger alpha values resulting in documents comprising many topics (ie, smaller alpha values suggest that documents comprise a small number of topics; [14]). Beta (also known as delta) influences how words create topics, with large values resulting in topics represented by a greater number of words (ie, smaller beta values suggest topics will be represented by fewer words; [14]). Once the LDA model is optimized, analysts can examine both the words and documents that are most probabilistically related to each topic to derive topic meaning and understanding of the larger textual data set.

**Figure 1:**
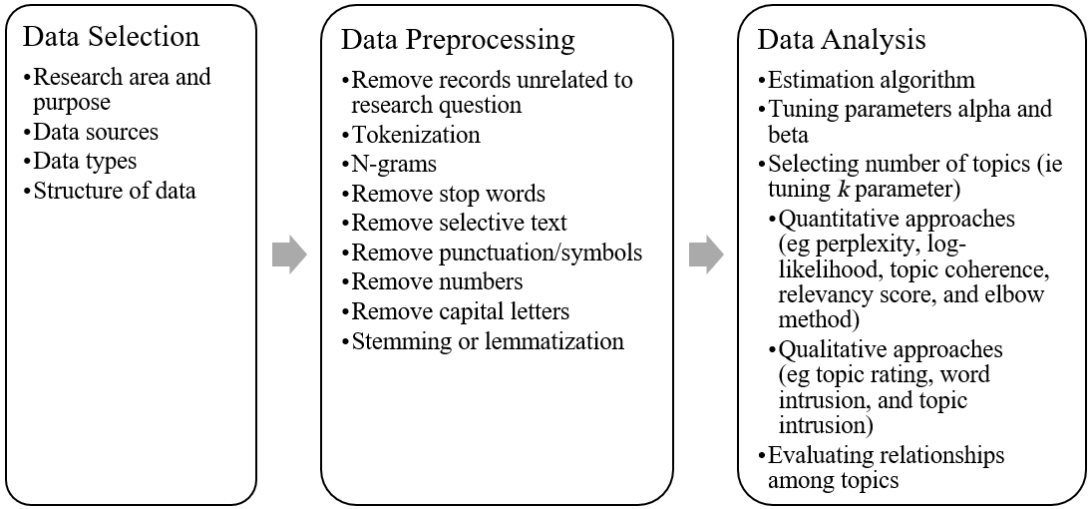
Summary of latent Dirichlet allocation (LDA) data selection, preprocessing, and analysis steps. Note: Tokenization is a required preprocessing step that ensures that the data are appropriately structured for analysis. All other preprocessing steps are optional.

As implied in the brief explanation above, training an LDA model is a complex task that involves decision-making and consideration of multiple factors that have the potential to influence the outcomes of the analysis. Several practical guides have been published [14-17] that broadly outline several different ways to approach LDA, using a variety of packages. Broadly, training an LDA model involves 3 major steps: *data selection*, *data preprocessing*, and *data analysis* (Figure 1). However, these are not prescriptive, and individual applications of LDA may involve iterations of these steps.

### Data Selection

The analyst must first make decisions regarding the textual data to be analyzed. The 4 major decisions in this step include determining (1) the research area and the purpose of the research being conducted, (2) the source of textual data, (3) the data types within these sources used for analysis, and (4) how data will be structured for analysis. Specifically, the research area and purpose of the research influences decisions made about the source of textual data (eg, social media, formal documentation, and scientific literature), the data types within that source that will be used for analysis (eg, original posts, comments, paragraphs, sentences, words, and other specific sections of text), and how these data will be structured (eg, by post, by user, by citation, and by paragraph) into documents (ie, units of text) for analysis.

### Data Preprocessing

Once a data set has been identified, the second major step involves preprocessing the text for analysis. Preprocessing is the process of preparing the data with the aim of increasing fidelity so that the results are meaningfully representative of the data [15,18] and relevant to the research question. Textual data sets have the potential to contain a substantial amount of noise and irrelevant textual information [18]. As outlined in numerous sources [15-17], textual data may require a range of general preprocessing steps depending on the research question. These may include, for example, converting to lower case, replacing entities (eg, people, places, and numbers) with placeholder using named entity recognition, and removal of punctuation and symbols, numbers, selective text that minimally contributes toward research questions and varies among studies, and stop words that are words thought to add no meaning to the data (eg, “and,” “it,” and “to”; [19]) and can be implemented using various stop word lists [20,21]. Furthermore, 2 processes of transforming words include stemming (ie, shortening words to a similar root form, without needing to have meaning; eg, “explore,” “exploratory,” and “exploration” into “explor”) and lemmatization (ie, transforming words to a canonical [lemma] form; eg, “explore,” “exploratory,” and “exploration” into “explore” [16]). Notably, although some research suggests using stemming or lemmatization cautiously because of the potential impact on results [16], the necessity of using this preprocessing step has also been called into question [22]. Finally, other preprocessing steps are undertaken to describe the way data are used in the analysis. Specifically, tokenization is when words are broken down into n-grams denoting single words (unigrams) or a series of words that are presented in the same order (2 words=bigram; 3 words=trigram [16]). Tokenization and n-grams are advantageous for disambiguating meaning in the context of surrounding words. For example, grouping “cognitive,” “behavioral,” “therapy” as a trigram allows researchers to observe how this construct contributes to a topic rather than how the individual words do.

### Data Analysis

Following preprocessing, the LDA analysis is typically conducted as the third step. There are 4 decision-making points during this step, including (1) the LDA estimation algorithm (eg, sampling approaches based on Markov Chain Monte Carlo [23,24], such as Gibbs sampling [11], and optimization approaches based on variational Bayes (VB) approximations [23,24], such as the variational EM algorithm [10]); (2) tuning parameters such as the alpha parameter [25], which influences how documents contribute to topics [14], and less importantly the beta parameter [25], which influences how words create topics [14]; (3) tuning the *k* parameter, that is, the process of selecting the number of latent topics that represent the data set, which can be done using quantitative (eg, perplexity [10], log-likelihood [14], topic coherence [26], relevancy score [27], and elbow method that is used to visually identify the optimal number of topics when plotting the results of quantitative metrics [28]) or qualitative approaches (eg, topic rating [29], word intrusion [30], and topic intrusion [30]); and (4) the process of evaluating relationships among topics.

LDA is a burgeoning approach with an increasing number of studies published in the psychological sciences. Several practical guides on LDA exist providing high-level advice, but they are inconsistent and not comprehensive. Therefore, the next steps in this research are to evaluate how LDA is being conducted by researchers in psychology and how this compares to synthesized advice from the existing guides, informing the development of best practice guidelines. Our aim was to conduct a scoping review to describe the methodological practices used in studies using LDA throughout the psychological literature. Scoping reviews focus on examining the nature of research activity and can be used specifically to survey how methodological approaches are implemented within an area of research [31-33]. Thus, a scoping review is particularly well-suited to examining the methodological practices of studies using LDA in psychology. Calvo et al [34] and Shatte et al [35] have previously conducted scoping reviews on broader machine learning techniques. Although these reviews examined the mental health literature and described different sources of textual data, they did not focus on the analytical decisions that were specific to LDA. This scoping review focuses on the key steps of *data selection*, *data preprocessing*, and *data analysis* as a framework to understand the methodological approaches being used in psychology research using LDA.

## Methods

### Transparency and Openness

This scoping review adhered to the PRISMA-ScR (Preferred Reporting Items for Systematic Reviews and Meta-Analyses extension for Scoping Reviews; [36]) and reports on search strategy, eligibility criteria, and data charting processes detailed in the following sections. This study was not preregistered.

### Search Strategy

Four electronic databases were searched using the following search strategy: “latent dirichlet” OR “topic* model*” OR “latent topic*.” MEDLINE Complete, CINAHL Complete, and EMBASE were searched up to April 15, 2020, with searches limited to the English language and research based on humans, with a peer-review limiter also applied to CINAHL Complete. PsycINFO was searched up to April 30, 2020, with English language and peer-review limiters applied.

### Eligibility Criteria and Selection of Sources of Evidence

Following the recommended practices for conducting scoping reviews [32], we used an iterative, team-based approach to finalize inclusion and exclusion criteria. Studies were included if they (1) were published in English, (2) were published in a peer-reviewed journal, (3) used LDA to analyze textual data, and (4) focused on a psychological construct or issue (eg, mental health issues, substance use, gender differences, and social issues such as same-sex marriage and environmental issues). Studies were excluded if they (1) were a commentary, letter, thesis, conference abstract or slides, or a methods paper; (2) used data that were not written words or words transcribed from speech (eg, genetic codes, mental health codes, and information derived from images); and (3) focused on constructs or issues that were nonpsychological in nature (eg, medical [37-40], marketing [4], and humanities [3]).

Titles and abstracts of all records were reviewed independently by 3 investigators (LJH, LMF, and GAO). All full-text records were assessed by a single investigator (LJH). In addition, 10% (71/712) of the articles were independently screened at the full-text level by another reviewer (LF or GAO) as part of the iterative process for refining inclusion criteria in accordance with recommended practices for conducting scoping reviews [32]. Disagreements during title and abstract screening and full-text assessment were resolved through discussion and consensus agreement by the research team.

### Data Charting Process, Data Items, and Synthesis of Results

A data charting (extraction) template based on common data selection, preprocessing, and data analysis steps was constructed and used to collate all relevant information from the included articles. The development of this data charting template was an iterative process that was continuously updated and refined during the data charting process.

In addition to study characteristics (ie, author, year, and journal of publication), the data charting process included the extraction of the (1) topic area (eg, mental health, depression, autism, self-harm, treatment, discrimination, and global climate) and purpose of research (ie, broadly what the study was aiming to achieve), (2) data sources (eg, social media, scientific literature, and formal documentation) and data types (eg, posts or comments, abstracts or titles, and selective words), (3) structure of the analyzed documents (eg, by user, post, patient, and citation), (4) data preprocessing steps conducted (eg, stop words, stemming, and lower casing), (5) LDA estimation algorithms used, (6) estimation parameters used, (7) relationships among topics, and (8) programs and packages used.

All charted data relating to study characteristics, topic area, purpose of research, data sources, and data types were tabulated according to the study, and all charted data relating to preprocessing and data analysis were tabulated according to the type of preprocessing step and methodological approach.

## Results

### Selection of Sources of Evidence

**Figure 2:**
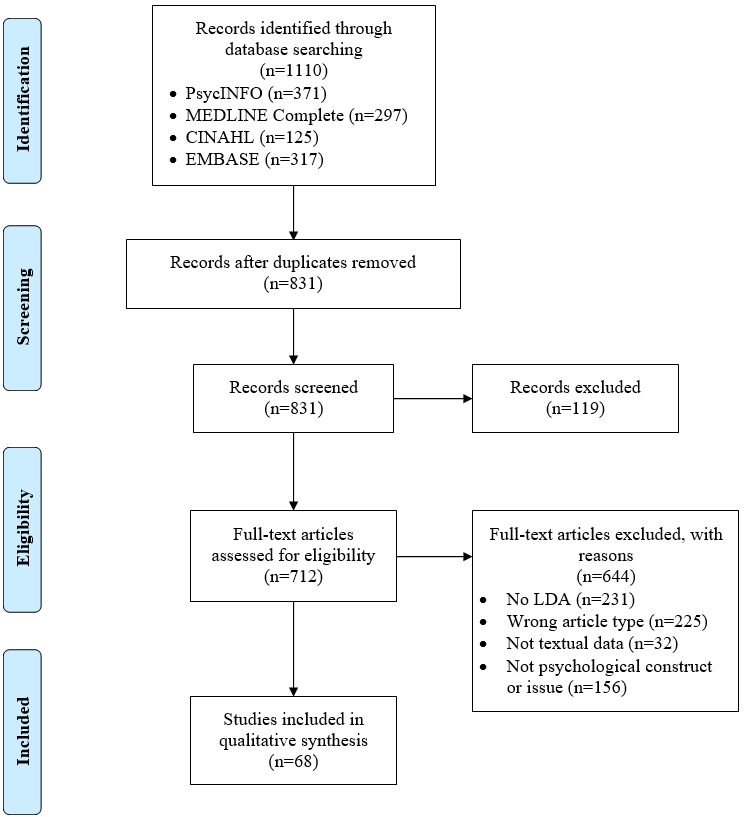
PRISMA (Preferred Reporting Items for Systematic Reviews and Meta-Analyses) flowchart detailing study inclusion and exclusion process [41]. LDA: latent Dirichlet allocation.

A PRISMA (Preferred Reporting Items for Systematic Reviews and Meta-Analyses) flow diagram of the systematic search results is shown in Figure 2 [41]. After removing duplicates (n=279), the search identified 831 articles for title and abstract screening. Of these, the full texts of 85.7% (712/831) potentially eligible articles were assessed, and 9.6% (68/712) of these articles were included in this scoping review.

### Characteristics of Sources of Evidence

Table 1 presents the characteristics of the included studies. The 68 studies that met the inclusion criteria were published between 2014 and 2020, with the application of LDA to psychological constructs increasing from 1 publication in 2014 to 11 in 2018 and 23 in 2019. A total of 13 articles were published in 2020 at the time of searching. Of the 55 different journals publishing these articles, the most frequent publication sources were the Journal of Medical Internet Research (7/68, 10%), PLOS One (3/68, 4%), and International Journal of Environmental Research and Public Health (3/68, 4%).

**Table 1:** Summary of study characteristics and data selection.

Author	Journal	Topic area	Purpose of research	Source of data	Data type nested within document level	Documents, n	Words per document (before or after preprocessing)
Abdellaoui et al [42]	*Journal of Medical Internet Research*	Substance use	Detect cases of noncompliance to drug treatment in patient forum posts	Social media; forum	Posts (escitalopram); post Posts (aripiprazole); post	Escitalopram=3649; aripiprazole=2164	NR^a^
Afshar et al [43]	*PLOS One*	Substance use	Identify subtypes in patients with opioid misuse	Formal documentation; clinical notes	Selective words; NR	NR	NR
Alam et al [44]	*Behaviour & Information Technology*	Social issues	Improve situational awareness of humanitarian organizations about disaster events	Social media; Twitter	Posts; NR	NR	NR
Barry et al [45]	*American Journal of Health Education*	Substance use	Examine advertising practices of alcohol brands	Social media; Twitter	Posts; NR	NR	NR
Bittermann and Fischer [46]	*Zeitschrift fur Psychologie*	Scientific topics	Identify hot topics in psychology	Scientific literature	Controlled keyword terms; citation	314,573	NR
Carpenter et al [47]	*Journal of Medical Internet Research*	Mental health	Assessing efficacy of internet well-being interventions	Social media; other—Happify	Free text response; task	NR	Mean 51.23 (before)
Carron-Arthur et al [48]	*BMC Psychiatry*	Mental health	Topics of discussion in mental health support groups	Social media; forum	Posts; post	131,004	Range 70-110 (after)
Chen et al [49]	*Journal of Medical Internet Research*	Substance use	Understanding electronic cigarette and hookah use	Social media; forum	Posts; NR	NR	NR
Choi and Seo [50]	*Issues in Mental Health Nursing*	Mental health	Provide an overview of depression of caregivers	Scientific literature	Abstracts; citation	426	NR
Choudhury et al [51]	*Strategic Management Journal*	Social issues	Investigate managerial cognitive capabilities and CEO^b^ communication	Other: interview transcripts	Interview transcripts; response to interview question	69	Mean 8234 (before; SD 3458)
Cohan et al [52]	*Journal of the Association for Information Science & Technology*	Mental health	Determining mental health based on indications for self-harm ideation	Social media; forum	Posts; NR	NR	NR
Feldhege et al [53]	*Journal of Affective Disorders*	Mental health	Investigate topics in a web-based depression community	Social media; forum	Posts and comments; user	20,037	NR
Franz et al [54]	*Suicide and Life-Threatening Behavior*	Mental Health	Identify self-injurious thoughts and behaviors and related themes on the web	Social media; forum	Posts; post	2355	Mean 43.21 (before; SD 42.99)
Gerber [55]	*Decision Support Systems*	Forensic	Predicting crime	Social media; Twitter	Selective tweets; neighborhood	NR	NR
Giorgi et al [56]	*Organization Science*	Social issues	Examine relationship between films and their legal environment via a cultural contingency perspective	Formal documentation; congressional hearings and annual reports; other; newspaper articles	Annual reports, congressional hearings, and newspaper articles; annual report, congressional hearing, and newspaper article	Annual report=84; congressional hearing=25; newspaper article=950	NR
Guo et al [57]	*PLOS One*	Social issues	Map the topic landscape of social class an inequality	Scientific literature	Selective words in titles, keywords, and abstracts; NR	NR	NR
Hemmatian et al [58]	*Behavior Research Methods*	Social issues	Demonstrate how change in the framing of same-sex marriage in public discourse relates to changes in public opinion	Social media; forum	Selective comments; NR	NR	NR
Hwang et al [59]	*Journal of Medical Internet Research*	Mental health	Analyze behavior patterns of emotional eaters	Social media; forum	Posts and comments; NR	NR	NR
Jaworska and Nanda [60]	*Applied Linguistics*	Social issues	Examine thematic patterns and their changes over time of corporate social responsibility reports in the oil sector	Formal documentation; social responsibility reports	Reports; NR	NR	NR
Jung and Suh [61]	*Decision Support Systems*	Mental health	Identifying job satisfaction	Other; company review website	Reviews; NR	NR	NR
Kagashe et al [62]	*Journal of Medical Internet Research*	Substance use	Understanding the use of medicinal drugs during seasonal influenza	Social media; Twitter	Posts; post	459,043	NR
Karami et al [63]	*Psychology of Violence*	Social issues	Understand experiences of sexism and sexual harassment in the workplace	Social media; Forum	Posts; post	2362	NR
Kee et al [64]	*Mindfulness*	Mental health	Identify topics relevant to mindfulness research	Scientific literature	Titles and abstracts; NR	NR	NR
Kigerl [65]	*Social Science Computer Review*	Social issues	Further understand cybercrime carding forums	Social media: forum	Posts; user	30,469	NR
Kreitzberg et al [66]	*Addictive Behaviors*	Substance use	Examine tobacco promotion	Social media; Instagram	Posts; post	4629	NR
Landstrøm et al [67]	*Sexualities*	Social issues	Explore how norms for appropriate behavior between parents and children are constructed	Other; various webpages	Posts; NR	NR	NR
Lee et al [68]	*Evolution and Human Behavior*	Evolution	Investigate mating-relevant self-concepts and mate preference	Social media; other—web-based dating profiles	Written descriptions; profile	7973	Mean 69.65 (before; SD 106.83)
Lee et al [69]	*European Child and Adolescent Psychiatry*	Mental health	Identify characteristics of Korean student suicide	Formal documentation; teacher reports	Selective words; NR	NR	NR
Liang et al [70]	*Journal of Health Communication*	Physical health	Identify associations between regional prevalence of obesity and overweight and regional information and social environments	Social media; Twitter	Tweets; NR	NR	NR
Liu et al [71]	*International Journal of Medical Informatics*	Social issues	Investigate gender difference in web-based health communities	Social media; forum	Post; NR	NR	NR
Liu et al [72]	*Journal of Biomedical Informatics*	Mental health	Determine symptom-based patient subgroups in mental illness	Formal documentation; clinical notes	Selective words; patient	1746	NR
Liu et al [73]	*Psychology, Health & Medicine*	Scientific topics	Identify hot topics in published review articles in clinical psychology	Scientific literature	Titles and abstracts; NR	NR	NR
Liu et al [74]	*International Journal of Environmental Research and Public Health*	Emotions; mental health; physical health	Study differences in the emotions of patients with physiological and psychological diseases	Social media; forum	Posts; post	17,891	NR
Lou et al [75]	*Journal of Interactive Advertising*	Social issues	Investigate how influencer vs brand-promoted advertisements affect consumer engagement, sentiment, and topics of comment	Social media; Instagram	Advertisement; NR	NR	NR
Louvigné and Rubens [76]	*Behaviormetrika*	Education	Classification of goal-based messages	Social media; Twitter	Tweets; learning goal	NR	NR
Magua et al [77]	*Journal of Women’s Health*	Social issues	Investigate disadvantages of being a woman in renewing grants	Formal documentation; summary statements	Summary statements; NR	NR	NR
McCoy [78]	*Psychosomatics: Journal of Consultation and Liaison Psychiatry*	Mental health	Map delirium literature	Scientific literature	Titles and abstracts; citation	3231	NR
Merrill and Åkerlund [79]	*Journal of Computer-Mediated Communication*	Social issues	Investigate how racism contributes to group discussion of immigration and how Facebook allows this	Social media; Facebook	Posts and comments; identical post	23,939	NR
Murdock et al [80]	*Cognition*	Development	Study exploration and exploitation trade-off	Other; nonfiction books	Books; NR	NR	NR
Oh et al [81]	*Journal of Counselling Psychology*	Scientific topics	Identify topics in *Journal of Counselling Psychology*	Scientific literature	Abstracts; NR	NR	NR
Pandrekar et al [82]	*American Medical Informatics Association annual symposium proceedings; American Medical Informatics Association symposium*	Substance use	Investigate opioid-related discussions	Social media; forum	Posts; NR	NR	NR
Pantti et al [83]	*European Journal of Communication*	Social issues	Investigate how racism is used in Finnish public debate	Social media: forum; other: news media content	Discussion forum content and news content; NR	NR	NR
Pappa et al [84]	*Journal of Medical Internet Research*	Physical health	Identifying factors associated with weight change	Social media; forum	Posts and comments; NR	NR	NR
Park and Conway [85]	*American Medical Informatics Association annual symposium proceedings; American Medical Informatics Association symposium*	Substance use; physical health	Track health-related discussions (ie, Ebola, e-cigarettes, influenza, and marijuana)	Social media; forum	Selective words from posts and comments; post	114,320,798	NR
Ray et al [86]	*Journal of Strategic Marketing*	Education	Explore values affecting behavioral intention in e-learning	Social media: Twitter; other: reviews	Review and tweets; review	Reviews=139,581; tweets=1442	NR
Ruiz et al [87]	*Attachment & Human Development*	Development	Investigate reflective functioning in fathers of children born preterm and at term	Other: survey data	Text response to 8 survey items; NR	NR	NR
Rumshisky et al [88]	*Translational Psychiatry*	Mental health	Predicting psychiatric readmission	Formal documentation; health records	Selective words; NR	NR	NR
Santos et al [89]	*Systems Research and Behavioural Science*	Social issues	Investigate the impact of social media and traditional media on democratic systems	Social media: Twitter; other: various webpages	Tweets and webpages; NR	NR	NR
Shahin and Dai [90]	*American Behavioral Scientist*	Social issues	Understand public engagement with global aid agencies	Social media; Twitter	Selective tweets; inbound data set	NR	NR
Shin et al [91]	*Frontiers in Psychology*	Education	Create distractor items	Other; open-source data set	Student responses; NR	NR	NR
Sieweke and Santoni [92]	*The Leadership Quarterly*	Social issues	Review research using natural experimental designs to infer causal relationships about leadership	Scientific literature	Abstracts; citation	1156	NR
Son et al [93]	*International Journal of Information Management*	Social issues	Investigate how Twitter’s representational features influence average retweet time and how effects differed based on type of disaster communication	Social media; Twitter	Tweets; NR	NR	NR
Sorour et al [94]	*Journal of Educational Technology & Society*	Education	Predict student performance	Other; student feedback	Selective words in comments; NR	NR	NR
Sperandeo et al [95]	*Frontiers in Psychiatry*	Mental health; personality	Investigate nature of research regarding personality and mental health	Scientific literature	Abstracts; NR	NR	NR
Szekely and Vom Brocke [96]	*PLOS One*	Social issues	Derive propositions for research and practice from corporate sustainability reports	Formal documentation; sustainability reports	Reports; NR	NR	NR
Törnberg and Törnberg [97]	*Discourse & Society*	Social issues	Analyzing discursive connections between Islamophobia and antifeminism	Social media; forum	Posts; user	576,801	1000 (before)
Tran et al [98]	*International Journal of Environmental Research and Public Health*	Mental health	Understand artificial intelligence application in the management of depressive disorders	Scientific literature	Abstracts; citation	NR	NR
Tran et al [99]	*Complementary Therapies in Medicine*	Mental health	Map mind-body interventions to improve quality of life	Scientific literature	Abstracts; NR	NR	NR
Turrentine et al [100]	*Journal of the American College of Surgeons*	Social issues	Examine gender differences in surgical residency applicants; recommendation letters	Formal documentation; letters of recommendation	Letters of recommendation; letter	332	Mean 404 (after)
Wang et al [101]	*BMC Public Health*	Substance use; mental health	Identifying topics about adolescent substance use and depression	Scientific literature	Abstracts; NR	NR	NR
Weij et al [102]	*International Journal of Consumer Studies*	Social issues	Discussion of attention to contemporary protesting artists among Western audiences	Social media; Twitter	Tweets; NR	NR	NR
Westmaas et al [103]	*Nicotine & Tobacco Research*	Substance use	Determine context of discussions surrounding cessation treatment for cancer survivors who smoke	Social media; forum	Posts; post	3998	NR
Wu et al [104]	*Journal of Educational Technology & Society*	Education	Investigate learner interest in open learning environments	Social media; other—Learning Cell Knowledge Community	Learning cell; learner	3538	NR
Yoon [105]	*Journal of the American Psychiatric Nurses Association*	Mental health	Identifying mental health needs for people with dementia	Social media; Twitter	Tweets and retweets; NR	NR	NR
Zhan et al [106]	*Journal of Medical Internet Research*	Substance use	Understanding how consumers and policy makers use social media to track e-cigarette–related content	Social media; Twitter and forum	Posts; NR	NR	NR
Zhao et al [107]	*International Journal of Environmental Research and Public Health*	Disability	Understand how autism-affected users use support groups on Facebook	Social media; Facebook	Interactions and content from 5 Facebook groups; NR	NR	NR
Zheng and Shahin [108]	*Information, Communication & Society*	Social issues	Examine social media use in pollical campaigns	Social media; Twitter	Tweets; NR	NR	NR
Zou [109]	*Expert opinion on drug safety*	Substance use	Analyze trends on drug safety	Scientific literature	Titles and abstracts; NR	NR	NR

^a^NR: not reported.

^b^CEO: chief executive officer.

### Data Selection

#### Research Area and Purpose

Table 1 shows that the most prominent areas of research were social issues (23/68, 34%; eg, racism, sexism, same-sex marriage, and global climate), mental health (19/68, 28%), and substance use (12/68, 26%). There was great variation among studies regarding the purpose of their research, which ranged from simply understanding behaviors (eg, e-cigarette and hookah use) and experiences (eg, sexism and sexual harassment) to assessing the efficacy of interventions (eg, internet well-being and mind-body interventions), identifying social discourse (eg, same-sex marriage, racism, and feminism), and analyzing trends (eg, drug safety).

#### Data Sources and Data Types

Table 1 highlights the key sources of the data used in LDA (Multimedia Appendix 1 [42-109] provides more details of data selection, data preprocessing, and data analysis) and the types of data used within these sources. The most common sources of data were social media platforms (35/68, 51%), which were most often derived from forums (eg, Reddit: 7/35, 20%) or microblogging platforms (eg, Twitter: 11/35, 31%; Facebook: 2/35, 6%; and Instagram: 2/35, 6%). Other social media sources included a knowledge community space (1/35, 3%) and web-based dating profiles (1/35, 3%). Studies typically sourced their data from one social media platform, with only 3% (1/35) of studies using multiple social media platforms as their source of data (ie, forum and Twitter). Of the studies that used data from forums and microblogging platforms, all indicated that they used some form of web-based posts (eg, original posts and comments) in their analyses. Some were explicit in that they specified the use of posts and comments or retweets (5/33, 15%), although some also included selective criterion (4/33, 12%; eg, selective comments containing negative and positive words or phrases [58] and selective words with specific term frequency–inverse document frequency scores [88]). Most studies, however, simply mentioned the use of “posts” or “tweets,” or “interactions online” or “discussion forum content” and did not describe their precise selection criteria (24/33, 73%).

Scientific literature was the next most common source of textual data (13/68, 19%), for which data were derived from searches of databases including Web of Science (5/13, 38%), MEDLINE (2/13, 15%), PubMed (2/13, 15%), and PSYINDEX (1/13, 8%). However, 23% (3/13) of the studies used scientific literature derived from specific journals. All studies using scientific literature specified the data used for analysis. Specifically, some studies only used data from abstracts (7/13, 54%), whereas others used data from titles and abstracts (4/13, 31%), controlled key terms (1/13, 8%), and selective words from titles, keywords, and abstracts (1/13, 8%).

Formal documentation was another common source of textual data (8/68, 12%), where data were derived from different forms of documentation such as sustainability, social responsibility, teacher reports (3/8, 37%), clinical notes (2/8, 25%), health records (1/8, 12%), summary statements (1/8, 12%), and letters of recommendation (1/8, 12%). These studies either used selective words from the documentation (4/8, 50%) or used the documentation in its entirety for analytic purposes (4/8, 50%).

Other uncategorized sources of textual data included nonfiction books (1/68, 1%), student feedback (1/68, 1%), survey data (1/68, 1%), interview transcripts (1/68, 1%), an open-source data set (1/68, 1%), a company review website (1/68, 1%), a web platform (1/68, 1%), and various webpages (1/68, 1%). The data types used in these studies are listed in Table 1.

Finally, although most studies used data from a single source, 6% (4/68) of the studies derived data from multiple sources. Of these, 75% (3/4) of the studies used data from social media microblogging platforms (eg, Twitter and forums) and other uncategorized sources including reviews, various webpages, and news media content. Moreover, of the 4 studies, 1 (25%) study used data from various formal documentation sources (eg, annual reports and congressional hearings) and an uncategorized source (newspaper articles).

#### Structure of Textual Data

Overall, 43% (29/68) of the studies reported how textual data were structured into documents for the purpose of analysis (Table 1). The remaining 57% (39/68) of the studies did not provide any methodological details on how the textual data were structured. Of the studies that reported on how they structured their data, those that derived data from social media commonly defined documents as individual posts (10/19, 53%) or a user’s history of posts (3/19, 16%). Studies that derived data from the scientific literature defined each document as text from individual publications (5/5, 100%), and studies that used data derived from formal documentation structured their data by patient (1/3, 33%), letter (1/3, 33%), or annual report or congressional hearing (1/3, 33%). Overall, 35% (24/68) of the studies reported sample sizes (ie, number of documents, which ranged from 69 documents to 114,320,798 documents (Median 3998, IQR 2164-30469). Finally, 10% (7/68) of the studies reported the number of words (or average number of words or range of words) per document (Median 90, IQR 60.44-702), and of those that did, 2 studies reported this value after preprocessing.

### Data Preprocessing

Overall, 86% (59/68) of the studies reported preprocessing their data. Table 2 highlights various preprocessing steps undertaken when preparing textual data for an LDA (Multimedia Appendix 1 describes preprocessing steps broken down by study). Specifically, the most frequently used steps included removing: stop words (46/59, 78%), punctuation, symbols or special characters (31/59, 53%), selective text (eg, hyperlinks, names, frequent words; 29/59, 49%), numbers (20/59, 34%), and invalid records (eg, records that do not provide relevant text; 17/59, 29%). Furthermore, 36% (21/59) of the studies undertook stemming or lemmatization, whereas 7% (4/59) studies explicitly stated that this step was not conducted [49,79,80,97]. Few studies reported conducting tokenization (15/59, 25%) and 15% (9/59) of the studies specified which n-grams were applied. Other preprocessing steps that were identified but less commonly used included removing capital letters, clearing whitespace, and correcting misspelled words (which can be conducted using automated spell checkers such as hunspell [110]). Overall, 10% (7/68) of the studies did not report data preprocessing, and 3% (2/68) of the studies indicated that data were preprocessed but provided no further details. Regarding the use of programs or packages for preprocessing data, 51% (35/68) of the studies did not comment on the tools used, 28% (19/68) highlighted the program or package used for all preprocessing undertaken, and 21% (14/68) specified the program or package for some preprocessing steps but not all (Multimedia Appendix 1).

**Table 2:** Summary of study engagement in data preprocessing, selection of *k*, and use of programs or packages.

Preprocessing steps (n^a^)	Selection of *k* (n)	Program; LDA^b^ package (n)
Stop words (46)	Quantitative approach (28)	Java; MALLET^c^ (15)
Punctuation, symbols, special characters (31)	Perplexity (11); [10]	R; Topicmodels package (13)
Selective text (29)	Harmonic mean of model log-likelihoods (5); [11]	R; MALLET package (2)
Stemming or lemmatization (21)	Topic coherence (4); [26]	R; stm package (1)
Numbers (20)	Log-likelihood (3); [14]	R; maptpx package (1)
Invalid records (17)	Kullback-Leibler divergence (3); [111]	R; KoNLP^d^ package (1)
Tokenization (15)	Jensen-Shannon divergence (3); [112]	R; dfrtopics package (1)
N-grams (9)	Exclusivity (1); [113]	R; LDA tuning package (1)
Unigrams (8)	Hierarchical Dirichlet process (HDP-LDA; 1); [114]	R; NR^e^ (4)
Bigrams (5)	Log Bays factor (1); [115]	Python; Gensim package (7)
Trigrams (1)	Per-document topic distributions (1); [62]	Python; LDA package (1)
Lower casing (16)	Topic probability (1); [116]	Python; Natural Language Toolkit package (1)
Whitespace (7)	Observing average *F*-measure (1); [94]	Python; NR (2)
Spelling (5)	Optimal_k function (1); [117]	Stata (2)
Unclear (2)	Minimization fit metric (1); [118]	Big text Tool (1)
NR (7)	*t*-distributed stochastic neighbor embedding (1); [91]	MeCab (1)
N/A^f^	Qualitative approach (10)	NR (17)
N/A	Quantitative and qualitative approach (5)	N/A
N/A	Topic coherence (4)	N/A
N/A	Perplexity (1)	N/A
N/A	Specificity (1); [119]	N/A
N/A	Kullback-Leibler divergence (1)	N/A
N/A	Sample size (1); [73]	N/A
N/A	Jensen-Shannon divergence (1)	N/A
N/A	Unclear (1)	N/A
N/A	NR (24)	N/A

^a^n: number of studies. Further details and references are provided in Multimedia Appendix 1.

^b^LDA: latent Dirichlet allocation.

^c^MALLET: Machine Learning for Language Toolkit.

^d^KoNLP: Korean natural language processing.

^e^NR: not reported.

^f^N/A: not applicable.

### Data Analysis

#### LDA Estimation Algorithms

As shown in Table 2, 75% (51/68) of the studies specified the program or package used to train the LDA model, with the most common implementation being Machine Learning for Language Toolkit (MALLET; 15/51, 29%), topic models in R (13/51, 25%), and Gensim in Python (7/51, 14%). Among the studies that used Gensim in Python, it was unclear whether Gensim’s implementation of LDA or Gensim’s LDA MALLET wrapper was used. Multimedia Appendix 1 provides the programs and packages used broken down by study.

Only 26% (18/68) of the studies explicitly reported the estimation algorithms used to train the LDA model (Multimedia Appendix 1). Most of these studies used a Gibbs sampling method (16/18, 89%). Overall, 74% (50/68) of the studies did not explicitly provide the estimation algorithms used. Of these 50 studies, 25 (50%) referred readers to algorithm-specific documentation (eg, the studies by Blei et al [10] for the variational EM algorithm and Griffiths and Steyvers [11] for Gibbs sampling), and 19 (38%) studies specified the programs and packages used for analysis, for which the default algorithms can be determined (eg, program or package documentation) and were likely used.

#### Selection of Alpha and Beta Parameters

Only 13% (9/68) of the studies (Multimedia Appendix 1) specified the selection of alpha and beta parameters. Specifically, the most consistently selected alpha parameters were 0.1 (3/9, 33%) and 50/*k* (3/9, 33%), and the most common beta parameter was 0.01 (5/9, 56%).

#### Selecting the Number of Topics (k Parameter)

An essential parameter that must be specified when training an LDA model is the number of topics. Table 2 highlights various approaches that have been applied to determine the optimal number of topics (Multimedia Appendix 1 provides an approach to determine the optimal number of topics broken down by study). Overall, the most common approaches were quantitative in nature (28/68, 41%). The most predominant approach was perplexity (11/28, 39%), which is a common method of evaluating model fit in LDA models [10,120], where models with lower perplexity are considered the best fitting. Another commonly used method for evaluating model fit was topic coherence (4/28, 14%), which allows for a comparison of topics by measuring the degree of semantic similarity among words that contribute the most to that topic [26]. Log-likelihood was also used (3/28, 11%), whereby the best-fitting model was considered to occur at the maximum log-likelihood value. These data suggest that perplexity and coherence remain popular approaches. Perplexity, which uses the log-likelihood, attempts to quantify how well an estimated model generalizes to a new data set. Although this is helpful for understanding the optimal number of topics in a data set, this approach can lead to uninterpretable topics; therefore, combining quantitative and qualitative measures should be used to assess the quality of the topics. Consequently, coherence metrics attempt to quantify the semantic relatedness of the words that are most strongly related to a topic. A model in which the *k* number of topics all have high coherence suggests that the topics will be more interpretable by researchers. Finally, a range of minimization and maximization fit metrics were used to determine the optimal number of topics (eg, harmonic mean of the model log-likelihoods, Kullback-Leibler divergence, and Jensen-Shannon divergence). A qualitative approach to determining the appropriate number of topics was used by 15% (10/68) of the studies, which involved using human judgment and researcher expertise to specify the number of topics. Furthermore, 7% (5/68) of the studies used a mixed methods approach to determine the optimal number of topics, and 1% (1/68) of studies suggested that LDA tuning was undertaken but did not specify how. Finally, 35% (24/68) of the studies did not report on how the optimal number of topics was determined.

### Evaluating Relationships Among Topics

Another consideration when training an LDA model is evaluating the relationships or overlap among topics (Multimedia Appendix 1). Overall, 85% (58/68) of the studies did not report the relationships among topics, and 7% (4/58) of these studies acknowledged this as a limitation of their research. The remaining 15% (10/68) of the studies that reported relationships among topics did so using hierarchical clustering analyses (3/10, 30%) or other study-specific methods including visualization techniques (4/10, 40%; eg, LDAvis).

## Discussion

### Principal Findings

Our aim was to conduct a scoping review to describe the methodological practices used in LDA studies throughout the psychological literature. We focused on the steps of data *selection*, *data preprocessing*, and *data analysis* as a framework to understand the methodological approaches being used in psychology research that use LDA. The inclusion of 68 empirical studies, all of which were published since 2014, demonstrates that psychology researchers are adopting LDA to draw insights from big data sets; however, we identified considerable variability in the reporting of the steps outlined in the available practical guides, ranging from 10% for the number of words per document to 86% for any preprocessing.

### Data Selection

#### Research Area and Purpose

The literature shows that the research areas evaluated using LDA included both narrow and broad foci. The areas of focus included behavioral, cognitive, and affective constructs, which can be categorized into the following research areas: mental health, social issues (eg, racism, sexism, same-sex marriage, and global climate), substance use, physical health, education, identification of scientific topics, human development (eg, exploratory behavior, and parenting), personality, emotions, forensics, disability, and evolution. Although the areas in which LDA has been applied fall within the range of research areas highlighted earlier, the purpose for which LDA is used in psychological research varies widely and includes understanding behaviors (eg, e-cigarette and hookah use) and concepts (eg, sexism), assessing the efficacy of interventions (eg, internet well-being and mind-body interventions), identifying social discourse (eg, same-sex marriage, racism, and feminism), and analyzing trends (eg, drug safety).

#### Data Sources, Data Types, and Structure of Data

The findings of this review demonstrate that the common sources of big data used in psychological LDA research are social media (eg, forums, Twitter, Facebook, and Instagram), scientific literature, and formal documentation (eg, reports, clinical notes, health records, summary statements, and letters of recommendation). Given that the content often examined in psychological research is of a sensitive nature (eg, mental health issues and personal experiences), it may be particularly relevant to consider the ethical implications of using publicly available data (eg, social media), which might be linked to a person’s identity. We encourage researchers to consult ethics boards when determining whether approval is needed to use such data, even if it is publicly available [121,122]. Furthermore, social media data can be more prone to grammatical errors and increased ambiguity (eg, owing to spelling errors and slang) compared with scientific literature and formal documentation and may require more in-depth preprocessing depending on the nature of the research question. Where required, social media data can be preprocessed using packages such as TweetTokenizer from the Natural Language Tool Kit [123]. Despite the potential challenges associated with social media data, most included studies (35/68, 51%) used social media data and were more likely to report the structure of textual data, and the length of included documents, compared with studies using scientific literature, formal documentation, and other uncategorized sources of textual data. However, the scientific literature was slightly more likely to report the sample size.

The results also demonstrate that LDA provides researchers with unique flexibility in selecting the type of textual data that can best answer their research questions. The selection of textual data for analysis plays an influential role in analysis outcomes; therefore, it is imperative that authors clearly specify their data inclusion and exclusion criteria to ensure reproducibility. For instance, researchers can use “original posts” alone, to obtain a broad overview of topics within a forum or group, or “original posts” plus the subsequent comments, which allows for the analysis of topics in discourse. Although all studies specified the type of data used for analysis, most studies that used social media data did not describe their precise data selection criteria and simply mentioned the use of “posts” or “interactions online.” Taken together, the literature demonstrates that more transparency is needed in reporting practices.

This review identified that less than half of the included studies (29/68, 43%) reported how textual data are structured into documents (ie, units of text). This is an extension of data-type selection decisions, as it is important to consider that the same set of selected data could be structured in multiple ways. This underreporting of document structures can have a potentially important influence on contextualizing results [16,124]. For example, the decision to use titles and abstracts as the set of data for analysis answers different research questions if documents are structured according to a citation or journal. Consequently, not reporting document structure clouds interpretation of any topics that have been derived. Furthermore, only a small number of studies reported sample size (ie, number of documents) and the length of the included documents. This minimal reporting may be linked to inconsistent evidence regarding the optimal sample size and length of documents for LDA. For instance, some evidence argues for a larger number of documents, as it may be theoretically impossible to identify meaningful topics from a smaller number of documents; however, it also suggests that there is a threshold whereby increasing the number will not affect the performance of the LDA [124]. Others indicate that the sample size is dependent upon theoretical and methodological considerations related to the research question [16]. In addition, documents that are too long or too short can produce results that are difficult to interpret [124]. In the context of short pieces of textual data (eg, Twitter posts), LDA may not perform well, as this approach assumes that there are multiple topics per document. Qiang et al [9] reviewed a range of alternative methods for the modeling of short text documents, which are more likely to comprise a single topic or have a lower ability to find co-occurrence patterns, although there is some evidence that LDA may also perform adequately with such texts [125]. Furthermore, Mehrotra et al [126] and Ito et al [127] identified that pooling textual data, and therefore making documents longer, leads to improved LDA topic models. In contrast, Sbalchiero et al [128] highlighted the potential effects of different length texts on results and complexities associated with topic modeling in long texts, which warrants further investigation. At this time, it is suggested that the best way to determine the appropriate length of a document is to observe the optimal model fit for samples of different text lengths [128] but to use other approaches such as qualitative or, as discussed, other NLP methods (see the study by Qiang et al [9] for a review of methods for analyzing short texts and a GitHub resource that supports the comparison of different algorithms for short text documents) when dealing with smaller texts. Given that the structure of textual data into documents, sample size, and document length may influence the LDA, it is important that researchers training an LDA model clearly report this information and that future empirical studies investigate how these factors may affect results.

### Data Preprocessing

In contrast to the suggested practices in existing guides, studies do not routinely report on data preprocessing steps, with 13% (9/68) of studies not reporting this. Given that preprocessing steps work to increase the fidelity of data to ensure that results are meaningfully representative of the data, this underreporting is problematic as it may influence analyses and compromise the interpretability and subsequent conclusions [129]. Studies that reported preprocessing of data typically conducted a common set of processes including removing stop words, selective text (eg, hyperlinks, names, and frequent words), punctuation or symbols, invalid records, and numbers, and conducting stemming or lemmatization. Furthermore, few studies have clearly reported the use of tokenization and n-grams; however, some studies have highlighted the use of tokenization but did not specify the n-grams applied. The overall scarce reporting of tokenization and n-grams even more so highlights that the focus of researchers has been on reporting preprocessing steps that aim to increase data fidelity (eg, stop words, punctuation or symbols, and numbers), and less so on reporting preprocessing steps that describe how data are organized for analysis (eg, tokenization and n-grams). A need for transparency surrounding the presentation of data is demonstrated by literature that suggests the suitability of both unigrams and bigrams [16]; however, methodological studies have suggested that bigrams may not improve categorization into topics [130]. This indicates the need for further research exploring best practices for preprocessing steps that describe how data are presented for analysis.

Although a number of studies chose to conduct stemming or lemmatization, some explicitly stated that to facilitate topic interpretation, this step was not conducted [49,79,80,97]. This is consistent with the findings of Yang et al [131], which suggest that although topic models with and without stemming provide similar results, the stemmed results may be more difficult to interpret. Similarly, other studies have suggested that stemming or lemmatization provides no meaningful improvement to the quantitative measures of model fit and has the potential to reduce topic stability [132]. Despite methodological studies erring toward not engaging in stemming or lemmatization [132], a number of studies in the psychological sciences continue to engage in this practice. We recommend that future studies reflect the necessity of stemming, given the existing evidence. In addition, research may evaluate the effects of different types of stemming or lemmatization [132,133] on the results. Future research should consider reporting results with and without stemming or lemmatization to demonstrate the potential effects on results, which can be used to inform best practice recommendations.

### Data Analysis

#### LDA Programs and Packages, LDA Estimation Algorithms, Selecting Alpha and Beta Parameters, and Selecting Number of Topics (k Parameter)

Although results revealed that many programs or packages were used to train the LDA model, among the most commonly used were Java, R, and Python. The open-source nature of each of these programs emphasizes that LDA is an accessible analysis type for researchers in psychology. As such, we recommend that these open-source programs continue to be used in practice; however, the different estimation algorithms used in each program should be considered.

The results indicated that Gibbs sampling was the most commonly used estimation algorithm. However, the selection of estimation algorithms is underreported (ie, reported by only 18/68, 26% studies), which may reflect a lack of understanding about the potential implications of selecting these algorithms. Although there are some conflicting methodological studies investigating these estimation algorithms (eg, see VB algorithms for evidence of appropriateness [134-136]), Gibbs sampling appears to be a generally robust approach as defined by better prediction of the optimal number of topics [11,137], as well as strong performance even when compared with newer algorithms [29]. Although decisions surrounding which estimation algorithms to use are often guided by practicality related to ease of implementation in analysis programs (ie, availability in widely used statistical packages), we suggest that the wide availability of Gibbs sampling within packages makes this approach a strong contender for use in psychological studies.

Although estimation algorithms are underreported, by mentioning the programs and packages used, it is possible for the reader to assume that the default algorithms highlighted in the associated documentation were likely used; however, packages often change default settings, and therefore, package and version numbers should be documented. Furthermore, although the literature has highlighted that programming languages provide default implementations of LDA [14], there is evidence suggesting that tuning of the alpha (but not beta) parameter is an important consideration [25]. Of the studies that specified alpha and beta, 78% (7/9) of studies overrode defaults and specifically tuned alpha (as 0.1 and 50/*k*) and beta (as 0.01).

A parameter that is tuned consistently throughout the literature is the *k* parameter, which is the selection of the number of topics derived from the model [138]. Throughout the psychological literature, it is evident that approaches used to determine the number of topics shift between qualitative and quantitative methodologies, which is reflective of inconsistencies in practical guides, where some advocate for the use of quantitative approaches (eg, perplexity, log-likelihood, and topic coherence; [14]), which can be conducted in multiple ways (eg, [139]), whereas others suggest using qualitative approaches (eg, human judgment and expertise [16]). Quantitative approaches are beneficial, as they can be faster, systematic, and can be validated using cross-validation [15], which is the process of randomly splitting data into portions and training the model on all but one of those portions and then validating the model on the remaining portion. Although qualitative approaches are more time consuming, they too can also be systematic and cross-validated. In addition, research has demonstrated that quantitative methods do not replace human judgment when deciding a model’s interpretability and that qualitative methods allow researchers to explore textual data in ways that model fit statistics do not [30]. Some human judgment approaches include *topic rating* that refers to viewing a topic and assigning a quality score [29], *word intrusion* that is the qualitative process of identifying out-of-place words within a topic to understand a topic’s coherence [30], and *topic intrusion* that evaluates a topic model’s distribution of documents into topics compared with human judgment of a document’s content [30]. There are benefits and drawbacks associated with these 2 different methods of determining number of topics, and Asmussen et al [15] posited that as akin to factor analytic models where interpretability of factors is as important as statistical model fit, the number of topics should be determined by a balance between a usable number of topics and appropriate model fit. Moving beyond topic modeling alone, the literature has begun to analyze textual data sets by conducting qualitative coding and comparing these results to topic models [54]. Considering the conflicting literature, it is interesting to note that very few studies in psychology have used a combination of these techniques [48,56,58,73,75]. Overall, there are various ways of determining the number of topics, and although several different authors have proposed recommended approaches [29,140,141], this is an area of ongoing research, as recommended approaches do not necessarily converge on the same value for *k* selected.

#### Evaluating Relationships Among Topics

The results indicate that evaluating the relationships among topics is not a common practice in LDA studies conducted in the psychological sciences. Specifically, evaluating the relationships among topics involves observing the overlap among topics and understanding how topics are similar or different. One of the ways this can be achieved is by visualizing topics using tools such as LDAvis in R [27] and pyLDAvis in Python [142]. Increased evaluation of the relationships among topics will allow for richer findings and the potential to identify unexpected links among topics.

### Limitations

This is the first study to evaluate the decision-making processes in psychological research studies that use LDA, thus providing researchers in this space with an introduction to some of the key considerations when training an LDA model. The findings from this review should be considered in light of certain limitations. First, the points of decision-making within the analytic pipeline discussed in this review should be considered by all researchers; however, there are other points of decision-making that fall within *data selection*, *data preprocessing,* and *data analysis* that were not included in this review, as they are discretionary depending on the research question. For example, stratified analyses by potential theoretical or methodological moderators can help identify whether there is consistency in latent topics identified across the strata [16], but the use of such moderators is dependent upon the research question being asked. In addition, researchers may find it useful to develop specific inclusion and exclusion criteria and extract data in a way that is driven by clearly developed working definitions. For example, researchers may develop dictionaries of words that can be used to identify relevant content, which are carefully constructed based on theoretical and expert opinions to reflect important aspects of the constructs of interest for a study [16]. However, it is important to consider that this may not always be appropriate because, for example, social media users may not use the same language as experts; therefore, the extracted data may not be representative. A data-driven approach may be useful in that it can capture a greater breadth of data; however, this can be time consuming. Second, of the studies that did not provide methodological details on how textual data were structured into documents (ie, units of text), inferences could be made for some of these studies based on the language used throughout the article. This may be considered a limitation, as this information was not included in the interpretation of results; however, we argue that this is an illustration of the primary issues surrounding the lack of reporting within this literature. Third, this review focused on mapping the literature rather than appraising its quality; therefore, it is important to note that the intensity of engagement with the 3 steps discussed throughout this review does not necessarily reflect the quality or accuracy of the results as they relate to the constructs under investigation. Fourth, this review only included studies that applied LDA to a construct or issue; therefore, studies providing insights into the LDA methodology have not been reviewed. Fifth, this review specifically focused on traditional applications of LDA rather than modifications thereof, as these are increasingly being used in psychology research. Although the LDA used by studies in this review was unsupervised, a supervised LDA approach [143] may be useful, particularly if the aim of the research is prediction. The supervised LDA permits the user to label each document with known properties that can be used for model fitting. Jacobucci et al [144] provided a recent example of supervised LDA, where they included information on whether the author of each document used in their model had a known history of suicide risk. The study by Šperková [145] provides further information about variations of LDA (eg, sentiment LDA and factorial LDA). Finally, this review focuses on one topic modeling approach rather than an overview of multiple topic modeling approaches. When conducting topic modeling, we encourage researchers to consider the suitability of other approaches; the study by Terragni et al [7] provides further information about other topic modeling approaches (eg, latent semantic analysis and embedded topic models).

### Conclusions

This review demonstrates that LDA is an accessible and flexible technique that provides researchers with the opportunity to reap the benefits of big textual data sets, and as such, we advocate for its continued use in the psychological sciences. Although some studies explicitly highlight engaging in data selection, data preprocessing, and data analysis, this was not always the case, thus reducing the capacity for reproducibility and evaluation of alignment with suggested practices. Therefore, we encourage researchers to be thorough and transparent in their reporting standards. To assist with reporting processes and to work toward best practice recommendations, we have developed an LDA Preferred Reporting Checklist (Table 3) outlining the key data selection, data preprocessing, and data analysis steps that researchers should report on where appropriate, or at the very least consider, when training an LDA model.

Furthermore, this review revealed that there is still an ongoing debate surrounding the necessity of certain preprocessing steps, the most appropriate estimation algorithms, and the most appropriate methods for determining the number of topics, with limited investigation into how these decisions may influence results. Given this, we recommend that future research be conducted across all stages of LDA to identify comprehensive and evidence-based best practice recommendations.

**Table 3:** Latent Dirichlet allocation (LDA) Preferred Reporting Checklist.

Section and topic			Item				Checklist item		Reported on page
**Data selection**									
	Research area and purpose	1				Develop research questions, aims, objectives, and hypotheses as to which topics are likely to emerge.			
	Research area and purpose	2				Consider the suitability of LDA; is this the most appropriate methodology to answer the research question (eg, consider if another topic modeling approach, especially for short texts, or traditional qualitative or quantitative approaches may be more suitable to the research question)?			
	Inclusion and exclusion criteria	3				State inclusion and exclusion criteria for textual data to be used in LDA analysis (eg, based on researcher-developed dictionaries or data-driven approaches)			
	Data sources	4				Indicate source of evidence (eg, social media, formal documentation, scientific literature, survey responses, and books) and comment on quality of writing. Consider ethical obligations associated with the use of a chosen data source.			
	Data types	5		Specify the data types (eg, original posts or comments, titles, abstracts, or keywords) from within data sources that will be used for analyses.					
	Structure of data	6		State the document level (eg, structured by citation, paragraph, post, and user).					
	Structure of data	7		Specify number of documents.					
	Structure of data	8		Specify length of documents (eg, range, mean, and SD).					
**Data preprocessing**									
	Program, package, and version	9		Specify the program, package, and version used for preprocessing and analysis.					
	Cleaning	10		List the preprocessing steps conducted (eg, punctuation, symbols and remove unrelated records, numbers, and whitespace).					
	Stop words and selective text	11		Specify which stop word lists were applied and whether selective text was removed (eg, frequently or infrequently used words, hyperlinks, and names).					
	N-grams and tokenization	112		Indicate the use of tokenization and specify the n-gram (eg, unigram, bigram, or trigram).					
	Stemming or lemmatization	13		Indicate use of stemming, lemmatization, or neither and provide a rationale for decision.					
	Stemming or lemmatization	14		Consider reporting results with and without stemming or lemmatization.					
**Data analysis**									
	Estimation algorithms	15		State estimation algorithm used for analysis (eg, Gibbs sampling and variational EM^a^ algorithm).					
	Tuning parameters (alpha, beta, and k)	16		Specify alpha (eg, 0.01), beta (eg, 0.1, 50/k), and *k* (number of topics) parameters.					
	Tuning parameters (alpha, beta, and k)	17		Detail iterative approach and specify metrics (eg, qualitative or quantitative such as coherence, perplexity, and log-likelihood) used to optimize parameters (ie, number of topics). Include an explanation of qualitative or quantitative cross-validation approaches.					
	Evaluating relationships among topics	18		Evaluate and comment on relationships among topics (eg, visualization of topic modeling).					
	Reporting results	19		Include examples of prototypical documents for each topic. If top words within topics have little coherence, use the label “uninterpretable” to describe those topics.					
Reproducibility: share deidentified data, code, and documentation			20		Publicly release deidentified data (when permitted), code, and documentation on platforms such as Open Science Framework to allow for reproducibility.				

^a^EM: expectation maximization.

## Appendix

Multimedia Appendix 1 Details of data selection, preprocessing, and analysis broken down by study.

## Abbreviations

LDA: latent Dirichlet allocation
MALLET: Machine Learning for Language Toolkit
NLP: natural language processing
PRISMA: Preferred Reporting Items for Systematic Reviews and Meta-Analyses
PRISMA-ScR: Preferred Reporting Items for Systematic Reviews and Meta-Analyses extension for Scoping Reviews
VB: variational Bayes
