# Supplementary material for: Examining Analytic Practices in Latent Dirichlet Allocation Within Psychological Science: Scoping Review
Source: J Med Internet Res. 2022 Nov 8;24(11):e33166. doi: 10.2196/33166 (PMC9682457; doi:10.2196/33166)
Supplement: Multimedia Appendix 1 [file jmir_v24i11e33166_app1.docx]

## Multimedia Appendix 1.

*Details of data selection, data pre-processing and data analysis broken down by study*

| Author, year | Source of data | Pre-processing programs; packages | Pre-processing steps | Method for selecting number of topics | Estimation Algorithm; alpha parameter; delta parameter | Analysis programs; packages | Evaluate relationships between topics |
| --- | --- | --- | --- | --- | --- | --- | --- |
| Abdellaoui et al., 2018 [42] | Social media: forum (Detec’t database; a database that collects messages from several French forums using a Web crawler.) | R; tm, SnowballC and slam packages | Convert to lowercase  Remove punctuation Remove stopwords Remove words used to build corpus (i.e., drug names)  Remove whitespace Stemming/lemmatization N-gram: unigram and bigram | Quantitative approach; Log Bays factor | Maximum a posteriori (MAP) algorithm; NR; NR | R; topicmodels and MAPTPX packages | Noted as limitation |
|  |  | NA | Remove invalid records |  |  |  |  |
| Afshar et al., 2019 [43] | Formal documentation: Clinical notes | Apache clinical Text Analysis Knowledge Extraction System (CTAKES) | Tokenization | Quantitative approach; topic coherence | NR; NR; NR | Python; Gensim package | NR |
| Alam et al., 2020 [44] | Social media: Twitter | NR | Convert to lowercase Remove stopwords Remove selective text Remove numbers Remove punctuation | NR | Reference to documentation; NR; NR | NR | NR |
| Barry et al., 2018 [45] | Social media: Twitter | NR | Remove stopwords Remove numbers  Remove whitespace  Remove punctuation  Stemming/lemmatization | NR | NR; NR; NR | R | NR |
|  |  | NA | Remove invalid records |  |  |  |  |
| Bittermann et al., 2018 [46] | Scientific literature: PSYNDEX database. Included articles, book chapters, reports and dissertations. | R; tm package | Remove whitespace Remove punctuation | Quantitative approach; log-likelihood | Gibbs sampling; 0.1; 0.01 | R; topicmodels package | Noted as limitation |
| Carpenter et al., 2016 [47] | Other: web platform (Happify) | Python | Tokenization | Quantitative approach; topic probability | Gibbs sampling; 5; NR | Java; MALLET | Inter-topic correlation matrix and scree plot |
|  |  | NA | Remove invalid records |  |  |  |  |
| Carron-Arthur et al., 2016 [48] | Social media: forum (Internet support group BlueBoard) | Java; MALLET | Remove stopwords | Quantitative approach; topic coherence and specificity Qualitative approach; semantic content | Reference to documentation; NR; NR | Java; MALLET | NR |
|  |  | NR | Remove contractions of common words |  |  |  |  |
| Chen et al., 2015 [49] | Social media: forums (Vapor Talk General E-Cig Discussion, Hookah Forum General Discussion, Vapor Talk Health & Safety, and the Stopsmoking subreddit) | Java; MALLET | Remove stopwords | Qualitative approach; semantic content | NR; NR; NR | Java; MALLET | NR |
|  |  | NR | Remove selective text |  |  |  |  |
| Choi et al., 2020 [50] | Scientific literature: Web of Science | R; tidytext package | Remove stopwords Tokenization | Qualitative approach; semantic content | Reference to documentation; NR; NR | R; topicmodels package | NR |
|  |  | R; qdap package | Spell check  Assessed for formatting errors |  |  |  |  |
|  |  | R; tm package | Stemming/lemmatization |  |  |  |  |
| Choudhury et al., 2019 [51] | Other: interview transcripts | NR | Remove selective text | Quantitative approach; Kullback-Leibler divergence, harmonic mean of model log-likelihoods, and Jensen-Shannon divergence | NR; NR; NR | R; topicmodels and ldatuning packages | Topic Entropy |
| Cohan et al., 2017 [52] | Social media: forum (ReachOut) | NR | N-gram: unigram and bigram | NR | Reference to documentation; NR; NR | NR | NR |
| Feldhege et al., 2020 [53] | Social media: forum (Reddit (r/depression)) | R; tm package | Convert to lowercase Remove selective text  Remove stopwords  Remove numbers Stemming/lemmatization | Quantitative approach; exclusivity and topic coherence | NR; Bayesian inference methods; Bayesian inference methods | R; stm package | NR |
|  |  | NA | Remove invalid records |  |  |  |  |
| Franz et al., 2019 [54] | Social media: forum (TeenHelp) | NR | Convert to lowercase  Remove numbers Remove punctuation Remove whitespace  Remove stopwords  Remove selective text | NR | Reference to documentation; NR; NR | R; topicmodels package | NR |
| Gerber, 2014 [55] | Social media: Twitter | NR | Remove stopwords  Remove selective text Tokenization | NR | Gibbs sampling | Java; MALLET | NR |
| Giorgi et al., 2019 [56] | Formal documentation; congressional hearings and annual reports Other; newspaper articles | NR | Spell check Tokenization Remove numbers Remove punctuation Stemiming/lemmatization Remove stopwords Remove selective text | Quantitative; Kullback-Leibler divergence and Jensen-Shannon divergence Qualitative; semantic content | Gibbs sampling | Java; MALLET | NR |
| Guo et al., 2018 [57] | Scientific literature: Web of Science | Python; Natural Language Toolkit (NLTK) package | Remove stopwords Remove punctuation | Quantitative approach; topic coherence | NR; NR; NR | Python; Gensim package | Noted as limitation |
|  |  | NR | Convert to lowercase  Stemming/lemmatization |  |  |  |  |
|  |  | NA | Remove invalid records |  |  |  |  |
| Hemmatian et al., 2019 [58] | Social media: forum (Reddit) | Python; Natural Language Toolkit (NLTK) package | Remove stopwords | Quantitative approach; perplexity and topic coherence Qualitative approach; semantic content | Reference to documentation; 0.1; 0.1 | Python | NR |
|  |  | NR | Convert to lowercase  Stemming/lemmatization  Remove selective text  Remove punctuation |  |  |  |  |
| Hwang et al., 2020 [59] | Social media: forum (Reddit (r/LoseIt)) | Python; Natural Language Toolkit (NLTK) | Remove punctuation Tokenization Stemming/lemmatization Remove stopwords  Remove selective text | NR | NR; NR; NR | Python; Natural Language Toolkit (NLTK) | NR |
| Jaworska et al., 2018 [60] | Formal documentation: Corporate Social Responsibility reports, environmental reports, social responsibility sections in annual reports. | Python; NR | Remove special characters | NR | Reference to documentation; NR; NR | Java; MALLET | NR |
|  |  | Java; MALLET | Remove stopwords |  |  |  |  |
|  |  | NR | Remove numbers |  |  |  |  |
| Jung et al., 2019 [61] | Other: Company review website (Jobplanet) | NR | Remove selective text Remove whitespace N-gram: bigram | Quantitative approach; perplexity | Reference to documentation; NR; NR | NR | Hierarchical clustering analysis |
| Kagashe et al., 2017 [62] | Social Media: Twitter | NR | Remove stopwords N-gram: uni-gram | Quantitative approach; per-document topic distributions | NR; 5; 0.01 | Java; MALLET | NR |
|  |  | NA | Remove invalid records |  |  |  |  |
| Karami et al., 2019 [63] | Social media: forum (www.everydaysexism.com; users post their experiences of workplace sexism and sexual harassment.) | Java; MALLET | Convert to lowercase  Remove numbers  Remove punctuation  Remove stopwords  Tokenization  N-gram: unigram | Quantitative approach; log-likelihood | NR; NR; NR | Java; MALLET | NR |
| Kee et al., 2019 [64] | Scientific literature: Web of Science (Science Citation Index Expanded, Social Sciences Citation Index, Arts & Humanities Citation Index, Emerging Sources Citation Index) | R; NR | Stemming/lemmatization Remove punctuation Remove stopwords | Quantitative; optimal_k function | Reference to documentation; NR; NR | R; topicmodels | NR |
|  |  | R; tm package | Remove selective text |  |  |  |  |
| Kigerl, 2018 [65] | Social media: forum (CSU, CardersForum, BitsHacking) | NR | Remove stopwords Remove selective text Remove punctuation | Quantitative; minimization fit metric, Kullback-Leibler divergence, harmonic mean of model log-likelihoods, Jensen-Shannon divergence, and perplexity using 5-fold cross validation | Gibbs sampling | R; topicmodels | NR |
| Kreitzberg et al., 2019 [66] | Social media: Instagram | NR | Remove stopwords Remove selective text Remove numbers  Remove symbols Stemming/lemmatization | NR | NR; NR; NR | R; topicmodels package | NR |
|  |  | NA | Remove invalid records |  |  |  |  |
| Landstrom et al., 2017 [67] | Other: various webpages | NR | Pre-processed | NR | Reference to documentation; NR; NR | NR | NR |
| Lee et al., 2019 [68] | Social Media: other (Online dating profiles) | R; tidytext package | Convert to lowercase  Remove punctuation  Remove symbols  Remove selective text Remove stopwords Remove incoherent translations  Tokenization  N-gram: unigram | Quantitative approach; perplexity using 5-fold cross validation | NR; NR; NR | R; topicmodels package | NR |
|  |  | R; hunspell package | Spell check |  |  |  |  |
|  |  | NA | Remove invalid records |  |  |  |  |
| Lee et al., 2020 [69] | Formal documentation: Student suicide case report | R; NR | Remove stopwords  Stemming/lemmatization | Qualitative approach; semantic content | NR; NR; NR | R; KoNLP package | NR |
| Liang et al., 2019 [70] | Social media: Twitter | R; tm package | Convert to lowercase Remove selective text Remove stopwords Remove punctuation Remove numbers | NR | NR; NR; NR | R; topicmodels | NR |
|  |  | NA | Remove invalid records |  |  |  |  |
| Liu et al., 2018 [71] | Social media: forum (Tianmijiayuan; is the largest and the most active online diabetes community in China.) | NR | Remove stopwords | Qualitative approach; semantic content | Gibbs sampling | R | NR |
|  |  | NA | Remove invalid records |  |  |  |  |
| Liu et al., 2019 [72] | Formal documentation: Clinical notes | NR | NR | NR | NR; NR; NR | NR | NR |
|  |  | NA | Remove invalid records |  |  |  |  |
| Liu et al., 2020 [73] | Scientific literature; Annual Review of Clinical Psychology, Clinical Psychology Review and Health Psychology Review | Python; Natural Language Toolkit (NLTK) package | Convert to lower case Remove punctuation Remove whitespace Remove selective text Remove stopwords Stemming/lemmatization | Quantitative; sample size and topic coherence  Qualitative; semantic content | NR; learned from the data; learned from the data | Python; Gensim package | LDAvis |
| Liu et al., 2020 [74] | Social media: forum (Baidu Post Bar; the world’s largest Chinese community, which allows users to search for different keywords to enter an online community associated with those keywords.) | NR | Remove selective text  Remove numbers  Remove punctuation | Quantitative approach; perplexity | Gibbs sampling | Python | NR |
| Lou et al., 2019 [75] | Social media: Instagram | NR | Convert to lowercase Remove punctuation Remove stopwords Stemming/lemmatization  Tokenization | Quantitative; topic coherence Qualitative; semantic content | Reference to documentation; NR; NR | R; topicmodels | NR |
| Louvigné et al., 2016 [76] | Social media: Twitter | NR | NR | Quantitative approach; perplexity | Gibbs sampling | NR | NR |
|  |  | NA | Remove invalid records |  |  |  |  |
| Magua et al., 2017 [77] | Formal documentation: National Institute of Health summary statements | Mallet | Remove stopwords | Quantitative approach; harmonic mean of model log-likelihoods | NR; NR; NR | R; Mallet package | NR |
|  |  | NA | Remove invalid records |  |  |  |  |
| McCoy, 2019 [78] | Scientific literature; Medline | Python; Natural Language Toolkit (NLTK) package | Tokenization Stemming/lemmatization | Quantitative approach; log-likelihood | Gibbs sampling | NR | NR |
|  |  | NR | Remove stopwords  N-gram: Unigram, bigram and trigram |  |  |  |  |
| Merrill et al., 2019 [79] | Social media: Facebook | NR | Removed stopwords | Qualitative; semantic content | NR; NR; NR | Java; MALLET | NR |
| Murdock et al., 2017 [80] | Other: Non-fiction books | NR; InPho Topic Explorer | Tokenization | Qualitative approach; semantic content | Reference to documentation; NR; NR | NR | NR |
|  |  | NR; Unidecode | Convert to lowercase Remove punctuation Remove numbers |  |  |  |  |
|  |  | Python; Natural Language Toolkit (NLTK) | Remove stopwords |  |  |  |  |
|  |  | NR | Remove selective text |  |  |  |  |
| Oh et al., 2017 [81] | Scientific literature: The Journal of Clinical Psychology | NR; TreeTagger | Stemming/lemmatization | Qualitative approach; semantic content | Reference to documentation; NR; NR | R; Mallet and dfrtopics packages | Topic Browser |
|  |  | NR | Remove selective text  Remove stopwords Remove punctuation Remove numbers Remove symbols |  |  |  |  |
|  |  | NA | Remove invalid records |  |  |  |  |
| Pandrekar et al., 2018 [82] | Social media: forum (Reddit (r/opiates)) | NR | N-gram: unigram | Quantitative approach; perplexity using 10-fold cross validation | Reference to documentation; NR; NR | Python; Gensim package | NR |
| Pantti et al., 2019 [83] | Social media: forum (Suomi24)  Other: news media content | NR | Remove stopwords Remove selective text Stemming/lemmatization | Quantitative; harmonic mean of model log-likelihoods | Reference to documentation; NR; NR | NR | NR |
| Pappa et al., 2017 [84] | Social media: forum (Reddit (r/LoseIt)) | NR | Remove stopwords  Remove symbols | NR | Gibbs sampling | NR | NR |
| Park et al., 2018 [85] | Social media: forum (Reddit) | NR | Convert to lowercase  Remove punctuation  Remove selective text | NR | Reference to documentation; NR; NR | Python; Gensim package | NR |
|  |  | NA | Remove invalid records |  |  |  |  |
| Ray et al., 2020 [86] | Social media: Twitter  Other: reviews (Coursera webpages) | NR | Remove stopwords  Remove punctuation | NR; LDA tuning | Gibbs sampling | R | NR |
|  |  | NA | Remove invalid records |  |  |  |  |
| Ruiz et al., 2020 [87] | Other: Survey data | NR; GermaLemma | Stemming/lemmatization | Quantitative approach; Jensen-Shannon divergence | NR; NR; NR | R; topicmodels package | NR |
|  |  | NR | Remove stopwords  Remove punctuation |  |  |  |  |
| Rumshisky et al., 2016 [88] | Formal documentation: The Partners HealthCare Electronic Health Records | Python; sci-kit learn package | Remove stopwords | NR | Markov Chain Monte Carlo chain; 50/k; 200/n | NR | NR |
|  |  | NR | Remove punctuation Remove numbers |  |  |  |  |
| Santos et al., 2019 [89] | Social media: Twitter Other: various webpages | NR | NR | NR | Reference to documentation; NR; NR | NR | NR |
| Shahin et al., 2019 [90] | Social media: Twitter | Java; MALLET | Remove stopwords Remove selective text | NR | Reference to documentation; NR; NR | Java; MALLET | NR |
|  |  | NR | Convert to lowercase  Tokenization |  |  |  |  |
| Shin et al., 2019 [91] | Other; open-source data set (Automated Student Assessment Prize) | Python; Natural Language Toolkit (NLTK) package | Convert to lowercase Stemming/lemmatization | Quantitative; perplexity, t-distributed stochastic neighbour embedding and Kullback-Leibler divergence | Gibbs sampling | Python; lda package | NR |
|  |  | NR | Spell check Remove numbers Remove selective text Remove stopwords |  |  |  |  |
| Sieweke et al., 2020 [92] | Scientific literature: The Leadership Quarterly | Python; spaCy | Remove stopwords Remove numbers Stemming/lemmatization Tokenization | Quantitative; topic coherence | Reference to documentation; NR; NR | Java; MALLET | Visualisation of topics |
| Son et al., 2019 [93] | Social media: Twitter | NR | Remove selective text N-gram: unigram and bigram | Quantitative; perplexity | Reference to documentation; NR; NR | NR | NR |
| Sorour et al., 2017 [94] | Other: Student feedback | NR | NR | Quantitative approach; observing average F-measure | NR; NR; NR | MeCab | NR |
| Sperandeo et al., 2020 [95] | Scientific literature: PubMed | R; tm package | Remove stopwords Remove selective text Remove numbers Remove punctuation | Quantitative approach; perplexity using 5-fold cross validation | Reference to documentation; NR; NR | NR | Hierarchical clustering analysis |
| Szekely et al., 2017 [96] | Formal documentation: Sustainability reports | Python; Natural Language Toolkit (NLTK) package | Remove stopwords  Stemming/lemmatization | Qualitative approach; semantic content | NR; NR; NR | Java; MALLET | NR |
|  |  | NR | Convert to lowercase  Remove symbols  Remove numbers Tokenization |  |  |  |  |
| Törnberg et al., 2016 [97] | Social media: forum (Flashback; one of the largest web forums in the world.) | NR | Remove stopwords | NR | Reference to documentation; 1.0; NR | Big Text Tool | Discursive network |
| Tran et al., 2019 [98] | Scientific literature: Web of Science | NR | NR | NR | NR; NR; NR | STATA; NR | NR |
| Tran et al., 2020 [99] | Scientific literature; Web of Science | NR | NR | NR | NR; NR; NR | STATA; NR | NR |
| Turrentine et al., 2019 [100] | Formal documentation: Letters of recommendation entered into the Electronic Residency Application Service (ERAS) | NR | Remove selective text Remove whitespace  Remove punctuation Spell check  Assessed for grammar and formatting errors | NR | NR; NR; NR | R | NR |
|  |  | NA | Remove invalid records |  |  |  |  |
| Wang et al., 2016 [101] | Scientific Literature: PubMed | Java; MALLET | Remove stopwords Remove selective text  Remove numbers | Qualitative approach; semantic content | Gibbs sampling; 0.1; 0.01 | Java; MALLET | Hierarchical clustering analysis |
| Weij et al., 2015 [102] | Social media: Twitter | NR | NR | NR | Reference to documentation; NR; NR | Java; MALLET | NR |
| Westmaas et al., 2017 [103] | Social Media: forum (Cancer Survivors Network (CSN); an online cancer survivor community) | NR | Remove selective text Remove stopwords  Remove numbers  Remove punctuation | Quantitative approach; harmonic mean of model log-likelihoods | Gibbs sampling | R; topicmodels package | NR |
| Wu et al., 2018 [104] | Social media: other (knowledge community) | NR | Remove stopwords | Quantitative approach; perplexity | Gibbs sampling; 50/k; 0.01 | NR | NR |
| Yoon, 2016 [105] | Social media: Twitter | NR | NR | NR | Reference to documentation; NR; NR | NR | NR |
| Zhan et al., 2017 [106] | Social media: Twitter and forum (Reddit, JuiceDB and Twitter) | Python; Natural Language Toolkit (NLTK) package | Remove stopwords  Stemming/lemmatization Tokenization | Quantitative approach; Hierarchical Dirichlet Process (HDP - LDA) | NR; NR; NR | Python; Gensim package | NR |
| Zhao et al., 2019 [107] | Social media: Facebook | Python; Natural Language Toolkit (NLTK) package | Cleansed and pre-processed | NR | Gibbs sampling; 50/k; 0.01 | Python; Gensim package | Interactive visualisation supporting rapid experimentation for interpretive hypotheses |
| Zheng et al., 2020 [108] | Social media: Twitter | NR | NR | NR | Reference to documentation; NR; NR | Java; MALLET | NR |
| Zou, 2018 [109] | Scientific literature: Medline-indexed and peer-reviewed journals (Current Drug Safety, Drug Safety, Expert Opinion on Drug Safety, and Pharmacoepidemiology and Drug Safety) | NR | Remove stopwords  Remove selective text  Remove numbers  Remove punctuation  Remove symbols | Qualitative approach; semantic content | NR; NR; NR | NR | Noted as limitation |
| *Note.* NR = Not Reported. NA = Not Applicable. | | | | | | | |
